# Using Assistive Health Technology to Assess Fall Risk Appraisal, Body Composition, and Physical Activity

**DOI:** 10.1093/geroni/igab046.100

**Published:** 2021-12-17

**Authors:** Ladda Thiamwong, Joon-Hyuk Park, Renoa Choudhury, Oscar Garcia, Maxine Furtado, Nicole Stallworth, Jeffrey Stout

**Affiliations:** 1 College of Nursing, University of Central Florida, Orlando, Florida, United States; 2 University of Central Florida, Orlando, Florida, United States

## Abstract

One-third of older adults have a discrepancy between perceived and physiological fall risks or maladaptive fall risk appraisal (FRA). Older adults who report high fear of falling and overestimate their physiological fall risk are less likely to participate in physical activity (PA). Limited data suggest the interrelation between fall risk appraisal, body composition, and objective measured PA. This cross-sectional study examines the feasibility of recruitment and acceptability of Assistive Health Technology (AHT), including the BTrackS Balance System (BBS), Bioelectrical Impedance Analysis (InBody s10), and ActiGraph GT9X Link wireless activity monitor. This study demonstrates the benefits of using AHT to study the associations among FRA, body composition, and PA in older adults. We hypothesize that rational FRA is associated with higher levels of PA and skeletal muscle mass and lower levels of percent of body fat and body mass index. Topics presentation included research protocol and preliminary results.

